# Lowered anti-beta1 adrenergic receptor antibody concentrations may have prognostic significance in acute coronary syndrome

**DOI:** 10.1038/s41598-019-51125-9

**Published:** 2019-10-10

**Authors:** Diana Ernst, Johan Westerbergh, Georgios Sogkas, Alexandra Jablonka, Gerrit Ahrenstorf, Reinhold Ernst Schmidt, Harald Heidecke, Lars Wallentin, Gabriela Riemekasten, Torsten Witte

**Affiliations:** 1 Department of Immunology and Rheumatology, Medical School Hannover, Hannover, Germany; 2 Uppsala Clinical Research Center, Uppsala University, Uppsala, Sweden; 3 CellTrend GmbH, Luckenwalde, Germany; 4 Department of Medical Sciences, Cardiology, and Uppsala Clinical Research Center, Uppsala University, Uppsala, Sweden; 5 Department of Rheumatology, University of Schleswig-Holstein, Lübeck, Germany

**Keywords:** Autoimmunity, Prognostic markers

## Abstract

Although several risk factors exist for acute coronary syndrome (ACS) no biomarkers for survival or risk of re-infarction have been validated. Previously, reduced serum concentrations of anti-ß_1_AR Ab have been implicated in poorer ACS outcomes. This study further evaluates the prognostic implications of anti-ß_1_AR-Ab levels at the time of ACS onset. Serum anti-ß_1_AR Ab concentrations were measured in randomly selected patients from within the PLATO cohort. Stratification was performed according to ACS event: ST-elevation myocardial infarct (STEMI) vs. non-ST elevation myocardial infarct (NSTEMI). Antibody concentrations at ACS presentation were compared to 12-month all-cause and cardiovascular mortality, as well as 12-month re-infarction. Sub-analysis, stratifying for age and the correlation between antibody concentration and conventional cardiac risk-factors was subsequently performed. Serum anti-ß_1_AR Ab concentrations were measured in 400/799 (50%) STEMI patients and 399 NSTEMI patients. Increasing anti-ß_1_AR Ab concentrations were associated with STEMI (p = 0.001). Across all ACS patients, no associations between anti-ß_1_AR Ab concentration and either all-cause cardiovascular death or myocardial re-infarction (p = 0.14) were evident. However among STEMI patients ≤60 years with anti-ß_1_AR Ab concentration <median higher rates of re-infarction were observed, compared to those with anti-ß_1_AR Ab concentrations > median (14/198 (7.1%) vs. 2/190 (1.1%)); p = 0.01). Similarly, the same sub-group demonstrated greater risk of cardiovascular death in year 1, including re-infarction and stroke (22/198 (11.1%) vs. 10/190 (5.3%); p = 0.017). ACS Patients ≤60 years, exhibiting lower concentrations of ß_1_AR Ab carry a greater risk for early re-infarction and cardiovascular death. Large, prospective studies quantitatively assessing the prognostic relevance of Anti-ß_1_AR Ab levels should be considered.

## Introduction

Coronary heart disease (CHD) remains a leading cause of death in developed countries^1^. Numerous risk factors for the incidence of CHD and acute coronary syndrome (ACS) have been well-validated, including hyperlipidemia, arterial hypertension, diabetes, smoking and family history of CHD^2^. Biomarkers for the prognosis post-ACS have been suggested, such as Growth Differentiation Factor-15 (GDF-15) or N-terminal pro-hormone brain natriuretic peptide (NT-proBNP)^3^, but currently no biomarkers stratifying for risk of re-infarction have been identified.

Recently, a group of antibodies against various G protein coupled receptors including Beta1 adrenergic receptors (ß_1_AR) were identified in healthy individuals, which is altered by age, sex and certain diseases. These antibodies exhibit effects on the receptor as novel ligands and are important for immune cell homeostasis. Both increased as well as decreased ab concentrations were associated with diseases and disease symptoms^4^.

Beta_1_AR is known to be expressed on endothelial cells, cardiomyocytes and fibroblasts. Existing data suggests that cardiac function may be influenced by regulating ß_1_AR^5,6^. ß_1_AR affects both myocardial contractility^7^ and heart rate regulation^8^. Current literature suggests that autoantibodies against ß_1_AR (ß_1_AR Ab) may influence the development of cardiomyopathy^9^, and in particular some evidence of prognostic benefit exists for ß_1_AR Ab in ischemic cardiomyopathy^10^.

The immunological mechanisms of ß_1_AR Ab however remain unclear, with conflicting reports of both protective or negative effects on ß_1_AR as well as agonistic and antagonistic functions of ß_1_AR^10–12^. It would appear that the function of antibodies against ß_1_AR may depends upon the exact binding site and clinical status at the time of measurement, as for example different effects of ß_1_AR Ab were described in healthy individuals compared to patients with cardiomyopathy^13^.

We previously demonstrated that significantly reduced anti ß_1_AR Ab concentrations were evident in patients with STEMI compared to other forms of ACS and healthy controls in a combined cohort of 212 ACS patients^14^.

To validate these findings, a large scale assessment utilising the PLATO cohort was performed, paying particular attention to the role of ß_1_AR Ab regarding the incidence of re-infarction and cardiovascular death in the first year post index ACS event. Given recent evidence that age may influence autoantibodies against G protein coupled receptors regarding antibody levels and function has been shown recently^4^. Earlier studies with autoantibodies in atherosclerosis and cardiovascular disease have been performed in younger CHD patients, below the age of 60 years, as differences regarding to the patients age in their quantity for example in MAZ-ab have been present^14–16^. For these reasons we were interested as well, if ß1AR Ab may have a different meaning in younger patients.

## Methods

Serum samples from the Platelet Inhibition and Patient Outcomes (PLATO) trial, involving over 18.000 patients with ACS, were analyzed. Inclusion criteria consisted of hospitalization with ischemic symptoms within the previous 24 hours for ≥10 minutes, ≥18 years of age and not being pregnant. Minimum of two of the following criteria were mandatory:

i. Compatible ECG changes (ST segment depression, transient ST-elevation ≥1 mm in two or more contiguous leads or new left bundle branch block)
ii. Raised biomarkers (Troponin I/T or CK/MB)
iii. Coronary angiography PCI planned.

and one of the following:

i. Age ≥60 years
ii. Previous MI or coronary by-pass, or known ischemic heart disease with ≥50% stenosis in ≥2 vessels
iii. Previous ischemic stroke, physician-confirmed transient ischemic attack, carotid stenosis (≥50%) or revascularization
iv. Diabetes mellitus
v. Peripheral vascular disease
vi. Chronic renal dysfunction.

Comprehensive details are included in the original publication of the PLATO trial^17^.

For the current analysis, STEMI and NSTEMI patients were selected chronologically, based on earliest recruitment to maximize follow-up data completeness. Anti-ß_1_AR Ab concentrations were measured in sera from all patients and compared to the primary outcome according to the same diagnostic criteria within the first year, with patient 1-year survival - both all-cause and cardiovascular-related - being the secondary outcome measures. Further sub-analysis, correlating individual antibody concentrations to documented cardiac risk factors, as well as sub-group stratification for patient age and ACS phenotype were additionally performed.

The current study was approved by the institutional ethics committees of the Medical School Hannover as well as the national and institutional regulatory authorities and ethics committees in Uppsala, Sweden. The methods were carried out in accordance with good clinical practice guidelines. All patients provided written informed consent before entering the PLATO trial.

### Detection of anti-β_1_AR Ab using ELISA

The commercially available CE-certified ELISA kit (CellTrend GmbH, Luckenwalde, Germany) for quantifying anti-β_1_AR Ab was used. ELISA Validation was performed according to the FDA’s “Guidance for industry: Bioanalytical method validation”^18^. Using native membrane extracts from cell lines overexpressing human β_1_AR to measure IgG antibodies against the transmembrane β_1_AR in its assumed physiological conformation^19,20^. Tests were performed with microtiter polystyrene plates with 96 wells, which were coated with human ß_1_AR overexpressing extracts from transfected Chinese Hamster Ovary cells. To maintain the conformational epitopes of the receptor we added 1 mM calcium chloride to each buffer and incubated duplicate samples of a 1:100 dilution at 48 °C for 2 hours. After washing, an incubation followed for 1 hour with a 1:20.000 dilution of horseradish peroxidase-labelled goat anti-human IgG (Jackson, Bar Harbor, ME, USA) for detection. Plates were incubated with a human monoclonal antibody against ß_1_AR (anti-ß_1_AR mab), to be able to obtain a standard curve (Fig. SD3). We generated a standard curve by standardizing antibody concentrations. For example (a) 6250 ng/ml anti-ß_1_AR mab for standard point 1, (b) 3125 ng/ml anti-ß_1_AR mab for standard point 2, (c) 1563 ng/ml anti-ß_1_AR mab for standard point 3, (d) 781 ng/ml anti-ß_1_AR mab for standard point 4 (e) 391 ng/ml anti-ß_1_AR mab for standard point 5 and (f) 195 ng/ml anti-ß_1_AR mab for standard point 6. Finally, the optical density was measured. All standard points were performed in duplicates. The threshold for anti-ß_1_AR mab detection was set at 100 ng/ml. Analysis of all patient probes, sera and plasma samples, was performed according to the described protocol by individuals who had no information regarding the patients’ characteristics.

### Statistical analyses

We used R version 3.3.2 (http://www.r-project.org) for all data analyses.

The concentrations of anti-β_1_AR Ab have been analyzed in groups separated by age (≤60/>60 years) and ACS entity. Continuous variables were tested using either the Kruskal-Wallis rank sum test (>2 groups) or the Wilcoxon rank sum test (2 groups). Categorical variables were evaluated using Fisher’s exact test. Kaplan-Meier survival curves were constructed with patients being stratified according to anti-ß_1_AR Ab concentrations ≤ median and >median, ACS phenotype (STEMI vs. NSTEMI) and (≤60 vs. >60 years). Patient outcomes and anti-ß_1_AR Ab were analyzed within Cox proportional hazards models, fitted to the different outcomes using either anti-ß_1_AR Ab and STEMI/NSTEMI anti-ß_1_AR Ab and age group (≤60/>60 years) and the respective interactions between them as variables.

To assess the relationship between anti-ß_1_AR Ab and conventional cardiovascular biomarkers the time of initial ACS presentation, scatterplots were constructed and given the continuous nature of both variables, Spearman rank correlations were performed.

## Results

### Cohort study

#### Patients demographics

Anti-ß_1_AR Ab concentrations were measured in sera of 399 NSTEMI and 400 STEMI patients enrolled in the PLATO ACS cohort. Patients were then stratified according to anti-ß_1_AR Ab concentration being either *greater than* or *less than or equal* to the median concentration. Sub-group baseline characteristics are summarized in Table 1. Whilst the majority of patients in both groups were male, no significance difference in gender (69.5% vs. 69.6%; p = 0.939) or age (median 61 [52.8–70.0] vs. 61 [53.5–70.0] years; p = 0.961) was evident between groups.

**Table 1:** Baseline characteristics of all patients included in the cohort analysis, grouped according to individual ß1-AR antibody titer compared to overall median titer value.

Demographics	N	ß_1_AR Antibody Titer (ng/ml)				p
		≤Median Titer		>Median Titer		
N		400		399		
Age, Years	799	61	[52.8–70]	61	[53.5–70]	0.961^a^
Female, N (%)	799	122	(30.5)	120	(30.1)	0.939^b^
Weight, kg	799	80	[70–90]	80	[71–90]	0.466^a^
Body Mass Index, kgm^−2^	799	27.7	[25.0–30.8]	27.9	[25.3–31]	0.402^a^
**Risk Factors**						
Habitual smoker, N (%)	799	152	(38.0)	164	(41.1)	0.386^b^
Hypertension, N (%)	799	250	(62.5)	251	(62.9)	0.942^b^
Dyslipidemia, N (%)	799	182	(45.5)	156	(39.1)	0.073^b^
Diabetes mellitus, N (%)	799	95	(23.8)	72	(18.0)	0.055^b^
**Clinical Factors**						
**Blood Pressure**						
- *Systolic*, mmHg	799	138	*[120–150]*	140	*[120–153]*	*0.469* ^a^
- *Diastolic*, mmHg	799	80	*[70–90]*	80	*[72–90]*	*0.113* ^a^
Heart Rate, min^−1^	799	73	[64–82]	74	[66–86]	0.039^a^
**Past Medical History**						
Angina Pectoris, N (%)	799	178	(44.5)	169	(42.4)	0.568^b^
Myocardial Infarction, N (%)	799	62	(15.5)	66	(16.5)	0.701^b^
Heart Failure, N (%)	799	8	(2.0)	21	(5.3)	0.014^b^
Percutaneous Coronary Intervention, N (%)	799	42	(10.5)	38	(9.5)	0.724^b^
Coronary Artery Bypass Graft, N (%)	799	17	(4.2)	11	(2.8)	0.336^b^
Transient Ischemic Attack, N (%)	799	7	(1.8)	7	(1.8)	1.000^b^
Ischemic Stroke, N (%)	799	13	(3.2)	10	(2.5)	0.673^b^
Peripheral Arterial Disease, N (%)	799	30	(7.5)	21	(5.3)	0.247^b^
Chronic Renal Disease, N (%)	799	12	(3.0)	14	(3.5)	0.696^b^
Beta-blocker, N (%)	799	311	(77.0)	301	(75.4)	0.453^b^
ACE inhibitor, N (%)	799	257	(64.2)	260	(65.2)	0.824^b^
Statin, N (%)	799	339	(84.8)	363	(91.0)	0.009^b^
Aspirin, N (%)	799	388	(97.0)	390	(97.7)	0.659^b^
Clopidogrel, N (%)	799	120	(30.0)	96	(24.1)	0.067^b^
**Acute Coronary Syndrome Phenotype**						
ST-Elevation MI, N (%)	799	159	(39.8)	241	(60.4)	0.001^b^
**Serum Biomarkers**						
Apolipoprotein A1, g/L	747	1.0	[0.9–1.2]	1.0	[0.8–1.1]	0.412^a^
Apolipoprotein B, g/L	747	0.8	[0.7–1.0]	0.8	[0.6–1.0]	0.740^a^
C-Reactive Protein, mg/L	747	2.7	[1.2–6.2]	3.5	[1.4–8.4]	0.018^a^
Cystatin, mg/L	747	0.7	[0.6–0.9]	0.8	[0.6–0.9]	0.315^a^
Interleukin-6 pg/ml	790	3.1	[1.8–6.5]	3.5	[2.0–7.1]	0.146^a^
NT-proBNP, pmol/L	799	391	[125–946]	336	[101–1007]	0.407^a^
GDF-15 ng/l	799	1485	[1126–2011]	1609	[1161–2210]	0.055^a^
GFR ml/min	747	120	[94–120]	120	[90–120]	0.237^a^
Troponin-I, μg/L	747	1.1	[0.2–3.8]	0.8	[0.2–4.1]	0.534^a^
Troponin-T, ng/L	799	173	[58–510]	194	[50–522]	0.938^a^

Higher titers were more prevalent among ST-elevation myocardial infarction patients, as well as those with known heart failure. An association with moderate C-reactive protein elevation was observed. No association with traditional cardiovascular risk factors were evident. Values are Median [Interquartile Range] unless otherwise stated. ACE: angiotensin converting enzyme. BNP: brain naturetic peptide. GDF-15: Growth Differentiation Factor-15. GFR: glomerular filtration rate. ^a^Wilcoxon test; ^b^Fisher’s exact test.

With regards to past medical history, a significantly higher incidence of heart failure was identified in patients with higher anti-ß_1_AR Ab concentrations was observed (2% vs. 5.3%; p = 0.014). Other than statin treatment (84.8% vs. 91.0%; p = 0.009), no significant differences in standard prophylactic treatments across all drug classes were identified within the cohort.

#### Anti-ß_1_AR Ab, ACS phenotype and rate of re-infarction

**Figure 1:**
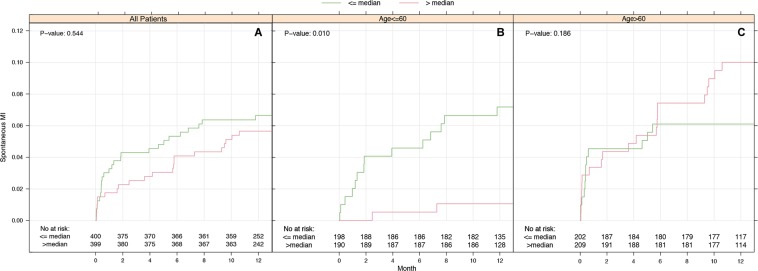
Kaplan-Meier curves for spontaneous MI over and under median ß1-AdrR-ab concentrations. Kaplan-Meier curves are stratified according to age groups. Number of events: all patients 48 (**A**), patients ≤60 years 16 (**B**) and patients >60 years 32 (**C**). ß1-AR ab below median was associated with a higher incidence of re-infarction in younger patients, particularly within the first 8 weeks. No differences were observed in patients >60 years (**C**) or all patients (**A**). P-values calculated using Cox-proportional hazards Score-Test.

**Figure 2:**
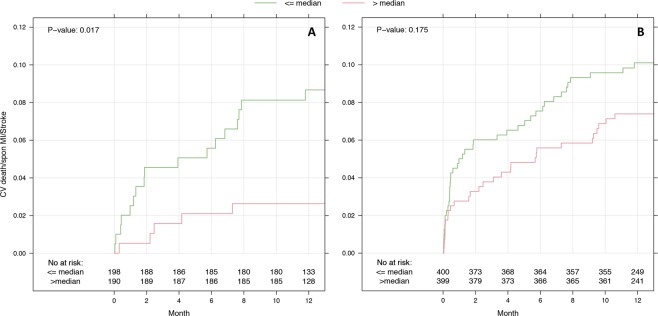
Kaplan-Meier curve summarizing all-cause cardiovascular mortality in the first 12 months after the index acute coronary syndrome with respect to ß1-AR ab level at original presentation for patients ≤60 years (**A**) and all patients (**B**). Once again, lower ß1-AR ab levels were associated with a higher incidence of fatal events in patients aged ≤60 years. In older patients, no differences were observed (p = 0.961, data suppl.). Number of events: 22 (**A**) und 69 (**B**). P-values calculated using Cox-proportional hazards Score-Test.

Comparing anti-ß_1_AR Ab levels between ACS phenotypes, a significantly higher proportion of STEMI patients exhibited concentrations of anti-ß_1_AR Ab above the median value (60.4% *vs*. 39.8%; p < 0.001). Those exhibiting lower anti-ß_1_AR Ab concentrations tended to have poorer cardiovascular outcomes in the subsequent demonstrated a higher incidence of cardiovascular deaths in the ensuing 12 months (n = 16, p = 0.067), although this failed to achieve significance. Likewise, no significant differences in the incidence of either stroke or myocardial re-infarction were observed within the entire cohort (p = 0.06), with sub-group analyses in patients < 60 years (n = 388) revealing that low anti-ß_1_AR Ab concentrations were associated with the incidence of re-infarction within 12 months (p = 0.010, Fig. 1). Considering all cardiovascular events collectively across these patients, patients with lower anti-β_1_AR Ab concentrations were associated with higher rates of adverse outcomes (p = 0.017, Fig. 2A).

In relation to spontaneous MI, patients with anti-ß_1_AR Ab concentrations ≤ median values appeared to incur a significantly higher number of events (n = 16; p = 0.010, Fig. 1) among younger patients ≤60 years. In older patients, spontaneous MI rates appeared largely independent of antibody concentration (n = 32; p = 0.186), Fig. 1C.

#### Anti-ß_1_AR Ab and conventional clinical risk factors

Between the stratified anti-ß_1_AR Ab concentration groups, no differences in the incidence or distribution of conventional risk factors across all patients were observed. Weak trends suggesting an association between lower anti-ß_1_AR Ab concentrations and increased incidence of diabetes mellitus (23.8% vs. 18.0%; p = 0.055) and dyslipidemia (45.5% vs. 39.1%; p = 0.073) were observed, levels (p = 0.055 and p = 0.073) in both groups were evident but neither achieved outright significance. Statin use was associated with higher anti-ß_1_AR Ab levels (p = 0.009).

Among patients ≤60 years, those exhibiting lower anti-ß_1_AR Ab concentrations had higher rates of diabetes mellitus (20.2 *vs*. 11.6%; p = 0.026) and were less likely to be receiving statins at study inclusion (83.8 vs. 91.6%; p = 0.021). Otherwise no differences in clinical risk factors in younger patients correlated with anti-ß_1_AR Ab concentrations (Table SD7).

#### Anti-ß_1_AR Ab and ACS biomarkers

Within the stratified anti-ß_1_AR Ab groups, the only serum biomarker demonstrating an inter-group difference was C-reactive protein, which was higher among patients with higher anti-ß_1_AR Ab concentrations (2.7 [1.2–6.2]mg/L vs. 3.5 [1.4–8.4]mg/L; p = 0.018). Further analysis, quantitively comparing the actual measured anti-ß_1_AR Ab concentrations with the respective serum biomarkers using the Spearman Rank Correlation test however confirmed that anti-ß_1_AR Ab did not correlate with any of the available biomarkers, apolipoprotein A1 and B, Cystatin, Troponin I and T, NT-proBNP, GDF-15, GFR and IL-6 (all coefficients R_s_ < ± 0.10; Supplemental Data Sheet, Fig. SD1 and Table SD8).

## Discussion

### Anti-ß_1_AR Ab levels in ACS

Across the entire cohort, empirical cumulative distribution functions of anti-ß_1_AR Ab concentrations revealed significantly higher values in STEMI than NSTEMI (p < 0.001), which retained significance following stratification for age > or ≤60 years (both p < 0.001; see Supplementary Data Sheet, Fig. SD2).

Overall, anti-ß_1_AR Ab concentrations across all 799 ACS patients failed to show any meaningful associations to either survival or re-infarction in the first year. Subgroup analyses using both unadjusted and adjusted Cox Regression Models demonstrated that younger patients with anti-ß_1_AR Ab concentrations ≤ median were less likely to remain free of either re-infarction (HR 0.14 [0.03–0.63]; p = 0.01) or any cardiovascular events (HR 0.30 [0.11–0.80]; p = 0.017), impacting inherently on event-free survival (Fig. 1). These findings corroborate to an extent earlier data, which suggested a worse outcome for ACS patients with low anti-ß_1_AR Ab levels^14^.

Furthermore, the current study associates lower Ab concentration with increased rates of early re-infarction, similar to trends seen in particular ACS sub-groups in our previous paper. To our knowledge, no biomarkers are routinely used in risk stratification for re-infarction, whilst only a few biomarkers such as GDF-15 or NT-proBNP are of proven prognostic relevance after ACS^3,21^, particularly in combination^22^. Future studies are needed, to explore if anti-ß_1_AR Ab could provide additional insight in such cases.

In contrast to previous data on anti-ß_1_AR Ab in ACS, which suggested that lower concentrations were found in STEMI compared to NSTEMI, both of which were markedly reduced compared to healthy controls^14^, the current study revealed higher concentrations in STEMI patients compared to NSTEMI. This may reflect, that STEMI is not automatically associated with greater injury or poorer worse outcome, as multiple additional confounders may have reasonably influenced mortality and prognosis^23–25^. STEMI patients are younger (FAST MI study 63.5 ± 13.8 (STEMI) vs. 68.1 ± 13.5 (NSTEMI)) and have a more transmural muscle damage, whilst NSTEMI patients are older, have more comorbidities and commonly a greater severity of coronary vascular disease. Therefore, NSTEMI patients have a lower early mortality but higher risk for long-term mortality compared to STEMI patients^26^.

Conceivably, our results regarding anti-ß_1_AR Ab concentrations in different ACS phenotypes may merely reflect demographic differences within the cohorts rather than any direct effect of anti-ß_1_AR Ab. It should be reiterated that the inclusion criteria for the PLATO trial excluded patients <60 years *without* known vascular comorbidities. Our original cohort was by contrast composed of particularly young ACS patients (NSTEMI median age 57 (48–61) years and STEMI 52(41–58) years) independent of comorbidities or known cardiac risk factors.

The results go along with the finding that anti-ß_1_AR Ab levels are independent of troponin or CK-values, suggesting no correlation to myocardial muscle damage.

Due to the lack of knowledge about the exact function of anti-ß_1_AR Ab, there are several possible explanations for these results. One could be a worse response to betablocker therapy. Nagatomo *et al*. have shown that patients with heart failure and evidence of anti-ß1AR Ab responded significantly better to betablocker therapy than patients with no increased anti-ß_1_AR Ab concentrations. Patients positive for anti-ß_1_AR Ab had for example a greater improvement in ejection fraction, greater changes of the left ventricular end-diastolic dimension and tended to have a better reduction of pro-BNP^27^.

A possible effect of betablocker therapy in our cohort is difficult to analyze as betablocker were taken in both groups, STEMI and NSTEMI, up to 80% of the patients before the ACS and nearly 100% after ACS. Even the following thesis is highly speculative, it could well be, that in earlier analyses patients with low anti-ß_1_AR Ab levels just did worse because they did not respond to ß-blocker treatment.

Another explanation for our results could be a stronger agonistic effect of anti-ß_1_AR Ab due to higher expression of ß_1_AR during and after an ACS^28^. This up-regulation of ß_1_AR may lead to a high binding of anti-ß_1_AR Ab and therefore falling levels in serum in ACS patients in general^14^. Suggesting a protective or blocking role of anti-ß_1_AR Ab on the receptors, the natural ligand adrenalin could stimulate the receptor stronger leading to vasoconstriction and worse outcome.

Statin use was associated with higher ab levels. Based on the role of statins in the prevention of infarction, high ab levels could be more beneficial.

### Risk factors and routinely used ACS biomarker

Based upon comparisons to conventional cardiac risk factors, no significant correlations to anti-ß_1_AR concentrations were observed. This may further supports the hypothesis of an additional autoimmune component to cardiovascular disease. Further research looking at the immunological aspects in RF-naïve ACS patients is needed. Whilst previous work has shown contradictory results regarding dyslipidemia^14^, we can show only trends towards a correlation with lower levels of anti-ß_1_AR Ab (p = 0.073).

It has to be noted, that the specific function of anti-ß_1_AR Ab may rely greatly on its binding antigen to the receptor, which could partially explain the contradicting results regarding the effect of anti-ß_1_AR Ab^29^.

Although levels for CRP were significantly higher in patients with high anti-ß_1_AR Ab 2.7 (1.2–6.2) mg/l versus 3.5 (1.4–8.4) mg/l (p = 0.018), values for IL-6 were not different, suggesting IL-6-independent associations for this observation.

### Anti-ß_1_AR Ab levels in congestive heart disease

Our results compliment the association of anti-ß_1_AR Ab with chronic heart failure, showing a significant increase of anti-ß_1_AR Ab in congestive heart failure (p = 0.014). Several anti-ß_1_AR Ab associated diseases are known to lead to chronic heart failure, such as dilated cardiomyopathy^30^, Chagas disease^31^ and atrial fibrillation^32,33^.

### Limitations

The aim of the current analysis was to validate the findings of our previous single center study in a large, well-characterized cohort. Despite the larger numbers of patients involved, this approach also introduced a number of further limitations to the current analysis.

The existing results suggested that the relevance of anti-ß_1_AR Ab in ACS was primarily in patients below the age of 60 years. Whilst the PLATO cohort included many such patients, there is an inherent inclusion bias against younger patients with no comorbidity. It is also unclear, what if any influence a previous cardiac event prior to the index event would have on antibody concentration. In this respect, limiting analysis to first presentation only may have been prudent.

Although the number of patients analyzed was high (n = 799), numbers of re-infarction, cardiovascular death and all-cause death were comparably low, hampering statistical analysis of subgroups.

Similarly, the cohort lacks a suitably matched healthy control group, we were not able to assess if the significance of anti-ß_1_AR Ab concentrations actually relates to the presence of an ACS or not.

The lack of understanding about the exact function of anti-ß_1_AR Ab is another main limitation. Therefore, the interpretation of our results remains speculative. Functional tests and longitudinal measurements are needed to further explore the functional influence and effect of anti-ß_1_AR Ab.

## Conclusion

Lowered titers of anti-ß_1_AR Ab in serum of ACS patients may be associated with higher risk for re-infarction or worse prognosis. These results have shown the greatest significance in patients below the age of 60 years. In this subgroup low anti-ß_1_AR Ab concentrations were associated with re-infarction (p = 0.01) and all cardiovascular events (p = 0.017) within 12 months after the index event. An association between low anti-ß_1_AR Ab and ACS compared to healthy controls and patients with atherosclerosis as well as a possible negative prognostic factor for survival and re-infarction has been shown recently^14^.

Larger cohorts are needed to finally confirm our findings. Furthermore functional analyses are necessary to understand the effect of anti-ß_1_AR Ab during and after ACS.

## Supplementary information

Supplementary Data
