# Supplementary material for: Lowered anti-beta1 adrenergic receptor antibody concentrations may have prognostic significance in acute coronary syndrome
Source: Sci Rep. 2019 Oct 10;9:14552. doi: 10.1038/s41598-019-51125-9 (PMC6787077; doi:10.1038/s41598-019-51125-9)
Supplement: Supplementary file 1 — Supplementary Data [file 41598_2019_51125_MOESM1_ESM.pdf]

Lowered anti-beta1 adrenergic receptor antibody concentrations may have prognostic significance in acute coronary syndrome

Diana Ernst[1], Johan Westerbergh[2], Georgios Sogkas[1], Alexandra Jablonka[1], Gerrit Ahrenstorf[1], Reinhold Ernst Schmidt[1], Harald Heidecke[3], Lars Wallentin[2,4], Gabriela Riemekasten[5*], Torsten Witte[1*]

[1]Department of Immunology and Rheumatology, Medical School Hannover, Germany
[2]Uppsala Clinical Research Center, Uppsala University, Sweden
[3]CellTrend GmbH, Luckenwalde, Germany
[4]Department of Medical Sciences, Cardiology, and Uppsala Clinical Research Center, Uppsala University, Sweden
[5]Department of Rheumatology, University of Schleswig-Holstein, Lübeck, Germany
*shared last authorship

Corresponding Author: Diana Ernst
E-mail: ernst.diana@mh-hannover.de

Supplementary Data Sheet:

Comparative Summary Of Adjusted & Unadjusted Cox Regression Analyses With Regard To Selected Patient Outcomes.

Variables included the adjusted analysis: Age(≤60/>60 years), STEMI, Gender, Statin at Presentation, Diabetes and Dyslipidemia.

	HR (CI 95%)	P
**All data**		
Unadjusted	0.72 (0.46 – 1.11)	0.140
Adjusted	0.71 (0.45 – 1.11)	0.135
**STEMI**		
Unadjusted	0.96 (0.51 – 1.79)	0.895
Adjusted	0.98 (0.52 – 1.83)	0.940
**NSTE-ACS**		
Unadjusted	0.55 (0.29 – 1.02)	0.060
Adjusted	0.51 (0.27 – 0.97)	0.039
**Age $\leq$ 60**		
Unadjusted	0.46 (0.22 – 0.97)	0.042
Adjusted	0.52 (0.24 – 1.13)	0.097
**Age $>$ 60**		
Unadjusted	0.93 (0.53 – 1.63)	0.796
Adjusted	0.80 (0.45 – 1.42)	0.446

Table SD1: CV Death, Myocardial Infarction or Stroke

	HR (CI 95%)	P
**All data**		
Unadjusted	0.72 (0.45 – 1.16)	0.175
Adjusted	0.68 (0.42 – 1.12)	0.131
**STEMI**		
Unadjusted	0.95 (0.48 – 1.91)	0.895
Adjusted	0.97 (0.48 – 1.96)	0.940
**NSTE-ACS**		
Unadjusted	0.54 (0.28 – 1.05)	0.068
Adjusted	0.50 (0.26 – 0.98)	0.043
**Age $\leq$ 60**		
Unadjusted	0.30 (0.11 – 0.80)	0.017
Adjusted	0.30 (0.11 – 0.83)	0.020
**Age $>$ 60**		
Unadjusted	1.01 (0.57 – 1.80)	0.961
Adjusted	0.89 (0.49 – 1.60)	0.698

Table SD2: CV Death, Reinfarction or Stroke

	HR (CI 95%)	P
**All data**		
Unadjusted	0.76 (0.46 – 1.26)	0.295
Adjusted	0.73 (0.44 – 1.23)	0.235
**STEMI**		
Unadjusted	1.03 (0.49 – 2.13)	0.943
Adjusted	1.07 (0.51 – 2.24)	0.856
**NSTE-ACS**		
Unadjusted	0.56 (0.28 – 1.13)	0.107
Adjusted	0.52 (0.26 – 1.05)	0.070
**Age $\leq$ 60**		
Unadjusted	0.27 (0.09 – 0.81)	0.020
Adjusted	0.28 (0.09 – 0.87)	0.028
**Age $>$ 60**		
Unadjusted	1.12 (0.61 – 2.04)	0.714
Adjusted	0.97 (0.52 – 1.80)	0.923

Table SD3: CV Death, Reinfarction

	HR (CI 95%)	P
**All data**		
Unadjusted	0.84 (0.48 – 1.48)	0.544
Adjusted	0.87 (0.48 – 1.56)	0.633
**STEMI**		
Unadjusted	1.06 (0.49 – 2.28)	0.883
Adjusted	1.09 (0.50 – 2.37)	0.824
**NSTE-ACS**		
Unadjusted	0.70 (0.30 – 1.66)	0.421
Adjusted	0.65 (0.27 – 1.54)	0.329
**Age $\leq$ 60**		
Unadjusted	0.14 (0.03 – 0.63)	0.010
Adjusted	0.16 (0.04 – 0.73)	0.018
**Age $>$ 60**		
Unadjusted	1.62 (0.79 – 3.32)	0.186
Adjusted	1.49 (0.71 – 3.11)	0.289

Table SD4: Spontaneous MI

	HR (CI 95%)	P
**All data**		
Unadjusted	0.56 (0.25 – 1.27)	0.163
Adjusted	0.47 (0.20 – 1.08)	0.077
**STEMI**		
Unadjusted	0.76 (0.19 – 3.05)	0.702
Adjusted	0.80 (0.20 – 3.26)	0.756
**NSTE-ACS**		
Unadjusted	0.39 (0.14 – 1.07)	0.067
Adjusted	0.36 (0.13 – 1.00)	0.051
**Age $>$ 60**		
Unadjusted	0.56 (0.22 – 1.42)	0.224
Adjusted	0.46 (0.18 – 1.20)	0.111

Table SD5: CV Death
(Sub-group <60yrs not performed due to small number of events)

	HR (CI 95%)	P
**All data**		
Unadjusted	0.76 (0.37 – 1.57)	0.457
Adjusted	0.62 (0.30 – 1.31)	0.210
**STEMI**		
Unadjusted	1.02 (0.29 – 3.60)	0.979
Adjusted	1.04 (0.29 – 3.74)	0.953
**NSTE-ACS**		
Unadjusted	0.53 (0.22 – 1.28)	0.157
Adjusted	0.49 (0.20 – 1.19)	0.116
**Age $>$ 60**		
Unadjusted	0.81 (0.36 – 1.82)	0.615
Adjusted	0.65 (0.29 – 1.50)	0.315

Table SD6: All Cause Mortality
(Sub-group <60yrs not performed due to small number of events)

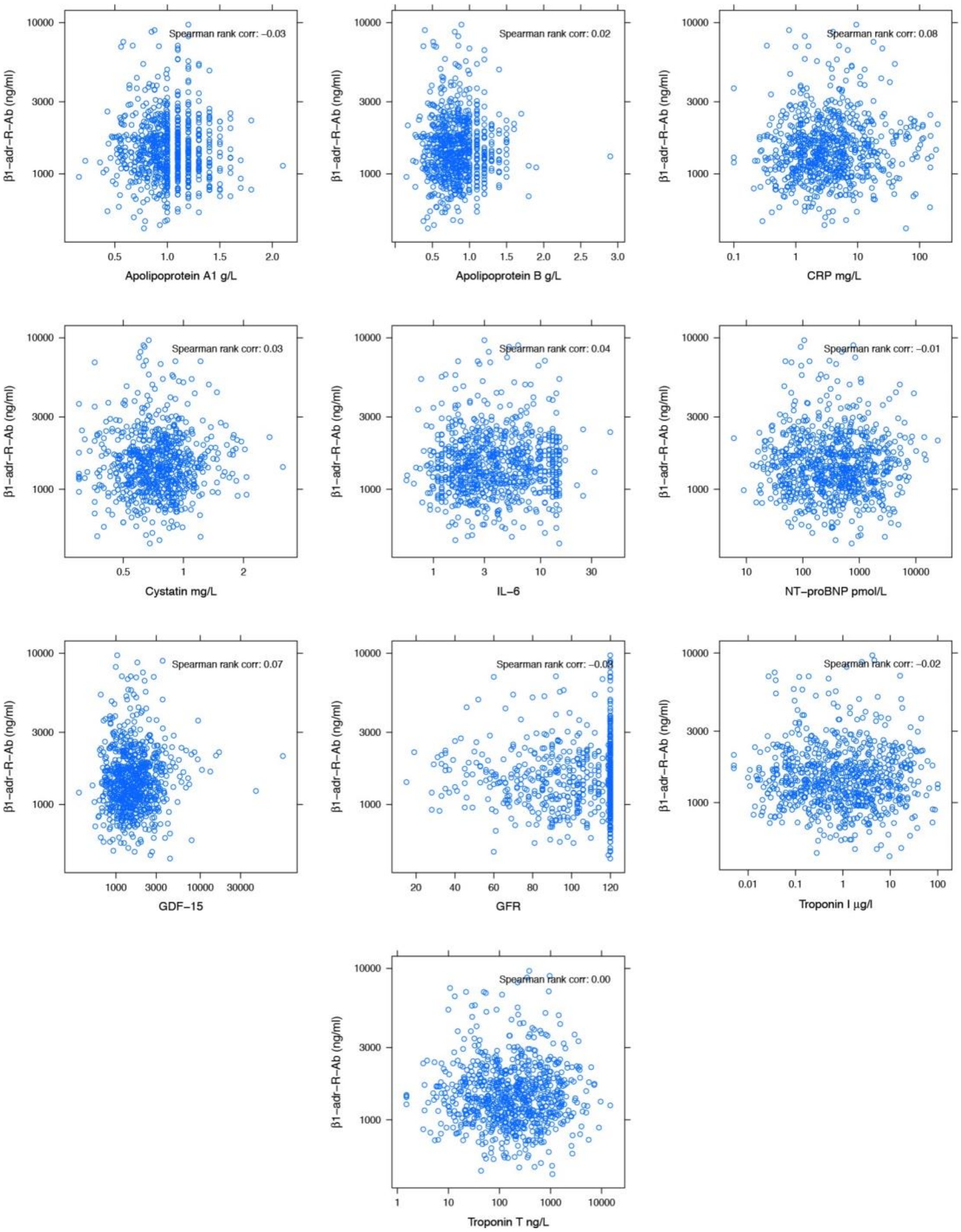

Fig SD1: Scatterplots comparing anti-$ß_1$AR Ab concentrations to various established cardiovascular biomarkers

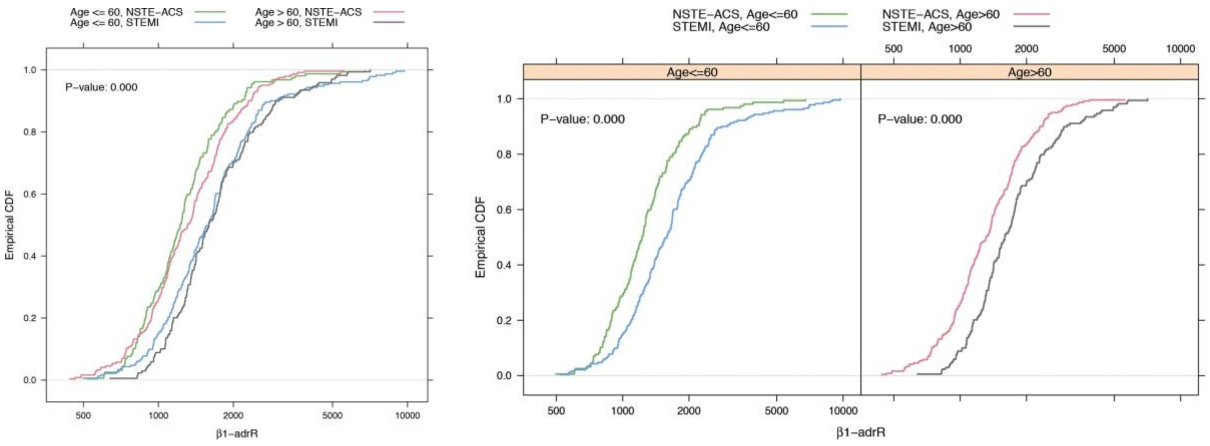

Fig SD2: Empirical cumulative distribution curves for anti-ß$_1$AR Ab concentrations across the entire cohort, stratified for patient age group and acute coronary syndrome phenotype. Legend: NSTE-ACS: Non-ST Elevation Acute Coronary Syndrome. STEMI: ST Elevation Myocardial Infarction

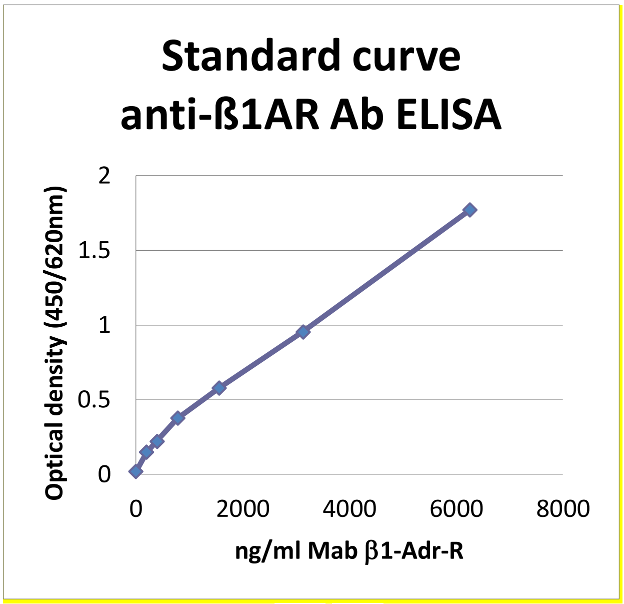

Fig SD3 : Standard curve of the anti-β$_1$AR Ab ELISA

Demographics	**ß$_1$AR Antibody Titer (ng/ml)**				p
	≤Median Titer		>Median Titer		
N	N = 198		N = 190		
Age, Years	52	[48.0-57.0]	53	[48.0-56.0]	0.506[1]
Female, N (%)	52	(26.3)	39	(20.5)	0.190[2]
Weight, kg	83	[72-92.8]	84	[74-93.0]	0.587[1]
Body Mass Index, kgm^{-2}	27.7	[25.0-31.2]	27.9	[25.5-30.9]	0.870[1]
**Risk Factors**					
Habitual smoker, N (%)	117	(59.1)	120	(63.2)	0.466[2]
Hypertension, N (%)	108	(54.5)	100	(52.6)	0.760[2]
Dyslipidemia, N (%)	88	(44.4)	72	(37.9)	0.216[2]
Diabetes mellitus, N (%)	40	(20.2)	22	(11.6)	0.026[2]
**Clinical Factors**					
Blood Pressure					
- *Systolic,* mmHg	*135*	*[120-150]*	*138.5*	*[120-152]*	*0.555[1]*
- *Diastolic,* mmHg	*80*	*[75 – 90]*	*80*	*[71 – 90]*	*0.937[1]*
Heart Rate, min^{-1}	73.5	[65 – 84]	74	[66 – 86]	0.239[1]
**Past Medical History**					
Angina Pectoris, N (%)	78	(39.5)	68	(35.8)	0.529[2]
Myocardial Infarction, N (%)	25	(12.6)	20	(10.5)	0.531[2]
Heart Failure, N (%)	4	(2.0)	6	(3.2)	0.536[2]
Percutaneous Coronary Intervention, N (%)	19	(9.6)	15	(7.9)	0.594[2]
Coronary Artery Bypass Graft, N (%)	6	(3)	1	(0.5)	0.122[2]
Transient Ischemic Attack, N (%)	2	(1.0)	0	(0.0)	0.499[2]
Ischemic Stroke, N (%)	2	(1.0)	4	(2.1)	0.441[2]
Peripheral Arterial Disease, N (%)	15	(7.6)	8	(4.2)	0.199[2]
Chronic Renal Disease, N (%)	1	(0.5)	3	(1.6)	0.363[2]
Beta-blocker, N (%)	145	(73.2)	138	(72.6)	0.909
ACE inhibitor, N (%)	129	(65.2)	114	(60.0)	0.345[2]
Statin, N (%)	166	(83.8)	174	(60.0)	0.021[2]
Aspirin, N (%)	192	(97.0)	184	(96.8)	1.000[2]
Clopidogrel, N (%)	60	(30.3)	41	(21.6)	0.064[2]
**Acute Coronary Syndrome Phenotype**					
ST-Elevation MI, N (%)	96	(48.5)	135	(71.1)	<0.001[2]

Tab. SD7: Summarizing clinical demographics and cardiovascular risk profile for all patients ≤60 years at enrolment. [1]Wilcoxon test; [2]Fisher's exact test.

	Correlation
	Rho
Apolipoprotein A1 g/L	−0.029
Apolipoprotein B g/L	0.020
CRP mg/L	0.078
Cystatin mg/L	0.027
IL-6	0.038
NT-proBNP pmol/L	−0.012
GDF-15	0.065
GFR	−0.031
Troponin I μg/L	−0.022
Troponin T ng/L	−0.001

Table SD8: Summary of Spearman Rank Correlation coefficients comparing anti-$ß_1$AR Ab concentrations to various established cardiovascular biomarkers
